# Exceptions to Broad Tissue-Specific Transcriptomic Interdependence: Searching for Independence in Expression of Genes

**DOI:** 10.3390/genes16091067

**Published:** 2025-09-10

**Authors:** Mikołaj Danielewski, Jarosław Walkowiak, Karolina Wielgus, Jan Krzysztof Nowak

**Affiliations:** 1 Department of Pediatric Gastroenterology and Metabolic Diseases, Poznan University of Medical Sciences, Szpitalna 27/33, 60-572 Poznan, Poland; mikolaj.danielewski@student.ump.edu.pl (M.D.); jarwalk@ump.edu.pl (J.W.); kwielgus@ump.edu.pl (K.W.); 2 Doctoral School, Poznan University of Medical Sciences, Bukowska 70, 60-812 Poznan, Poland

**Keywords:** gene, expression, transcriptome, uncorrelated, independent, tissue, blood, ileum, correlation

## Abstract

Background: Correlation of genes within tissues has attracted much attention. In contrast, genes that are INDependent In Expression (INDIE) remain poorly understood, even though they may represent tissue admixtures, reflect new regulatory mechanisms, either transcriptional or post-transcriptional, and contribute to biomarkers or machine learning algorithms. We hypothesised that INDIE genes can be found, may remain uncorrelated across tissues, and replicate within tissues in external datasets. Methods: Biweight midcorrelation was calculated for each gene against all other genes with sufficiently high expression in the given tissue from the GTEx dataset v8, along with the means of absolute values of obtained correlation coefficients. The threshold for gene designation as INDIE was both absolute (r) and relative (Z-score), while the threshold for external validation in the whole blood (four datasets) and the ileum (two datasets) was relative. Results: Only one gene, *RPL13P12*, was INDIE in all the analysed GTEx tissues, but it did not replicate in the external datasets. In contrast, *HIST1H2AD* and *TMEM176B* were not only INDIE in GTEx whole blood but also replicated in all four external datasets, despite their heterogeneity. Moreover, *ACAT2* replicated in both external ileal datasets. The haemoglobin gene *HBB* belonged to most widespread INDIE genes in various GTEx tissues and was validated in an external ileal dataset, pointing towards the importance of tissue heterogeneity in bulk samples. Conclusions: A set of genes exhibiting independent expression patterns across various tissues of GTEx was described. Results for each tissue are made available. Even though many findings can be explained by tissue heterogeneity, some results point towards interesting mechanisms of gene expression regulation.

## 1. Introduction

Gene expression is known to correlate across a group of bulk samples, forming co-expression modules [1], which are often involved in specific biological functions. There are, however, other potential reasons for gene expression correlation, such as gene location within the genome, activity of transcription factors, the unique properties of tissue and proportions of cells within a mixture. While much research focuses on broad gene co-expression and its link with traits, not much attention is paid to individual genes which appear to be expressed in the smallest groups or even individually.

Genes which are INDependent In Expression (INDIE) could be defined as the least correlated with the rest of the transcriptome, such as—for the purpose of this study—genes with expression levels that have an absolute correlation coefficient r < 0.2 and r < 5th percentile (within given tissue). Using both parameters allows for the selection of the least correlated genes, even in tissues where the absolute correlation is generally low. We speculate that such INDIE genes might be drivers of important but more narrow biological processes. Because of their unique characteristic, INDIE genes might be informative for complex biomarkers, including those that are based on machine learning, minimising collinearity that otherwise commonly affects transcriptomic models. Genes that are independent in expression can contribute to AI models by helping to maximise the information value of datasets. They could be perceived as noise and removed, because they may be relatively few (depending on the tissue), but in fact, they contain information. In terms of information theory, it could be speculated that they represent entropy—compressing the whole dataset without them would be disproportionately easy, compared with that without other genes that are contained in co-expression clusters. Of course, random data can also be more difficult to compress, but the information (independent data) content of those genes is interesting to consider. The fact that a gene is uncorrelated does not determine its biological importance. However, while there may be redundant genes with biological importance for an AI model to select from, an uncorrelated gene is not redundant—it will either be included in the model (especially if it reflects biology in a useful way) or will need to be rejected, or—in a small study—it may correlate with biological characteristics by chance and contribute to overfitting. Therefore, the fact that these genes are less prone to collinearity problems puts them in a separate category, the properties of which are worth considering in a time heavily influenced by AI technologies. Thus, they can be carriers of independent information within the gene expression profile. The conceptual framework for such approach is not unique, and is equally reflected in the concept of entropy from information theory (how much redundant data can be compressed) [2] and in practical applications, such as independent component analysis [3]. The biology underlying INDIE genes within transcriptomes is interesting because of their behaviour, which may fuel two divergent hypotheses: that silencing of such outlying genes would have no severe impact (when they could be used as drug targets) or that knock-out would be critical because of no redundancy (exposing critical molecular mechanisms). If there are, however, no reasonably INDIE genes in the transcriptome, then the whole gene expression profile needs to be perceived as an overlap of modules and a set of information that is practically much less dimensional that the total number of typically expressed genes would suggest.

The interrelatedness of gene expression is dependent on the tissue or cells that are considered, but it can contain information about relationships between cell types and their functional states or even synchronised cooperation of many cells, such as in functional brain networks [4]. Bulk sequencing data are rich in correlation relationships, even if they lack specificity due to the aforementioned inclusion of all cells within a tissue sample. In contrast, single-cell RNA-seq (scRNA-seq) data are very specific but sparse, making detection of genome-wide correlative patterns more difficult [5]. Low gene expression in scRNA-seq also requires the use of imputation techniques, which might introduce additional bias. If imputation is dependent on gene expression correlations, its accuracy will rely on the strength of correlations. For example, Mitić et al. [6] proposed a new method for feature selection in scRNA-seq data that reconciles correlative analysis with the sparse and compartmentalised nature of scRNA-seq data. As this is a challenge, and because of the increased sensitivity, bulk sequencing is a viable option for the search for gene expression correlations, while the issue of the cellular composition of samples is taken into account.

In fact, a tissue may naturally contain an admixture of cells that are not typically considered as its constituents (e.g., migrating leukocytes). This could be viewed as contamination and could provide one explanation for the weakest gene expression correlations. But those seemingly intruding cells may also constitute an inherent part of the given tissue, and to answer many questions, they should be analysed in tandem. Furthermore, caution in biological interpretation of the transcriptome is required, because it is dynamic, undergoing synthesis, degradation, and epitranscriptomic regulation, and it does not directly reflect the amount of protein product. This is reflected by environment-dependent functions of cells exhibiting similar transcriptomes [7].

Nevertheless, transcriptomes offer great potential for furthering the understanding of mechanisms of health and disease and for biomarker discovery [8]. One of the largest and most successful transcriptomic studies was led by the Genotype-Tissue Expression (GTEx) Consortium, which supplied a wealth of data on the relationships between genomic variation and gene activity [9]. The GTEx provided the largest and most comprehensive available transcriptomic dataset to date, the essential part of which was obtained through bulk RNA sequencing, covering a broad variety of tissues obtained post-mortem that are not available from healthy donors (e.g., tissues of the brain). We hypothesised that INDIE genes can be identified across GTEx tissues and that there are INDIE genes in GTEx whole blood and terminal ileum data that may be independently replicated in external datasets.

The rationale behind this study is that genes most independent in expression from the largest number of other genes may possess unique information that is not accessible in co-expression modules that are typically related to large biological processes. Because positive verification of the lack of existence of a correlation is not a typical approach, we rely on custom methods that are most easily interpretable—including demonstration of a replicable lack of medium or strong correlation with other genes from the dataset. The results are likely to include genes that are truly the most independent in expression in the human transcriptome, but functional validation and interpretation of their value cannot be fully accomplished in silico. Multiple causes of transcriptomic independence can be imagined: shifts in cell proportions, cellular compartment localization (e.g., nuclear), housekeeping and constitutive expression, and stochastic expression, as well as unique mechanisms of gene expression regulation (genomic, epigenomic; potentially related to evolution) and rapid variability in response to fast-changing stimuli that does not affect other genes.

## 2. Methods

The Genotype-Tissue Expression (GTEx) transcriptome dataset v.8 was downloaded from the consortium repository (at www.gtexportal.org (accessed on 24 August 2025)) in transcript per million (TPM) normalisation, together with sample data. Only samples with an RNA integrity number (RIN) > 7 were retained. Tissues with less than 100 samples per GTEx-specific tissue descriptor (labelled “SMTSD”) were excluded from further analysis. Median expression (in TPM) for each gene across tissues was accessed via the GTEx portal to calculate the quantile allowing for the retention of at least 10,000 genes (out of 56,200) on average. This threshold of expression was 6 TPM. Therefore, for each tissue, the corresponding samples were selected, and the resulting dataset was further filtered to include only genes with a median gene expression (in this given tissue) of at least 6 TPM. Other genes were discarded because of insufficient expression potentially increasing the risk of containing a large number of uninterpretable values or no expression, translating to false positive findings in this study. The dataset was then transposed, log2-transformed, and scaled. Individual column names were assigned by pasting gene names and Ensembl transcript identifiers. Biweight midcorrelation (bicor) was calculated between all the genes (pairwise-complete observation) using the fast *bicor* function from the WGNCA package [10].

Means of absolute values of correlation coefficients were calculated for each gene. A high mean value would therefore indicate a greater number of stronger individual correlations with other genes, regardless of whether they were positive or negative. Genes with mean absolute values of correlation coefficients r < 0.2 were considered as the least correlated with other genes. This is related to the fact that approximately 0.2 is the weakest correlation that can be detected as statistically significant with 100 samples. With 100 samples (we only included tissues that had more than 100 samples, and half of the tissues had more than 200 samples), a significance level of 0.05, and a statistical power of 0.8, the correlation we could reliably detect as significant was lower than r = 0.276. Thus, we assumed that correlations lower than r = 0.2 were not likely to be meaningful and therefore could be argued to represent genes that were independent in expression. Values of r below 0.2 or 0.1 are considered by various authors to indicate negligible correlation or, at best, weak correlation [11]. However, the distribution of absolute correlation of gene expression differs between tissues. Because of the need to process a large number of results with optimum specificity, we chose to additionally ensure that genes with the weakest correlation in GTEx had mean absolute correlations not only below 0.2 but also below the 5th centile for the given tissue (Z-score −1.645). This increased result specificity in tissues with general low level of absolute correlation. Therefore, due to the need for analysis of GTEx, we defined INDIE genes as having a mean absolute correlation < 0.2 and mean absolute correlation Z-score < −1.645.

In order to identify cross-tissue INDIE genes, the number of tissues where a gene was INDIE was divided by the number of tissues where its expression level was sufficiently high for analysis. Thus, we calculated the percentage of tissues in which each gene was INDIE.

**Figure 1:**
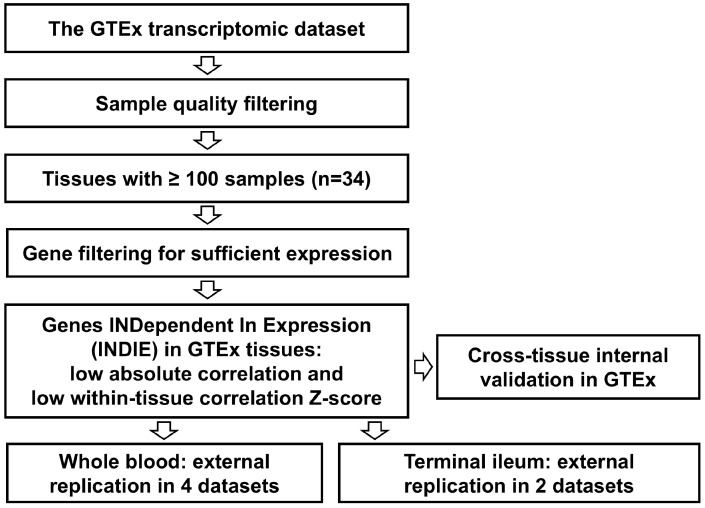
Study design. GTEx—Genotype-Tissue Expression.

External validation of the main findings was attempted in the whole blood and the terminal ileum, because of the availability of data and our ongoing research interest in the blood and the gut (relevant to inflammatory bowel disease, IBD). The following datasets were used: one by our collaborators from IBD-CHARACTER which included patients with IBD and healthy controls [8] (E-MTAB-11349 at BioStudies/ArrayExpress), one by Kumar et al. from a study of patients with interictal migraine (E-MTAB-13397), one by Gupta et al. from a study of patients with SARS-CoV-2 [12] (E-MTAB-10022), one by Rosenheim et al. from a study of patients with SARS-CoV-2 infection [13] (E-MTAB-12993), one by Momozawa et al. from persons undergoing screening colonoscopy in the CEDAR (Correlated Expression and Disease Association Research) project [14] (E-MTAB-6667), and one by Haberman et al. from the RISK cohort (Pediatric RISK Stratification Study) of patients with IBD [15] (GEO GSE57594). The data were TPM- or CPM-normalised, and the threshold of median expression for analysis was ≥6, as in GTEx, with the exception of the CEDAR dataset, which was obtained using microarrays, practically increasing the threshold for validation because of methodological differences. In CEDAR, genes with median expression values above the highest-expressed control probe were included. Means of absolute values of correlations of genes were calculated as in GTEx. However, because of the differences between datasets (bias towards lower or higher correlation values overall), we chose the Z-score corresponding to the 5th percentile as the threshold for validation (Z-score < −1.645). This threshold was applied to means of between two and four Z-scores in the external datasets for whole blood transcriptomes and the terminal ileum [16]. Additional information on individual genes were drawn from inspection of single-cell data as allowed by the UCSC (University of California, Santa Cruz, CA, USA) Cell Browser [17]. The study design is illustrated in Figure 1.

## 3. Results

### 3.1. GTEx Correlations in Tissues—Descriptive

**Figure 2:**
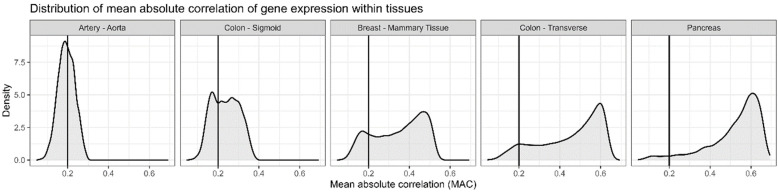
Density plots illustrating distribution of the mean absolute correlation (bicor) of gene expression within tissues: while overall some tissues demonstrated low correlation (**left**), others were highly correlated internally (**right**). Mean absolute correlations illustrated in the figure were calculated for each gene separately.

**Figure 3:**
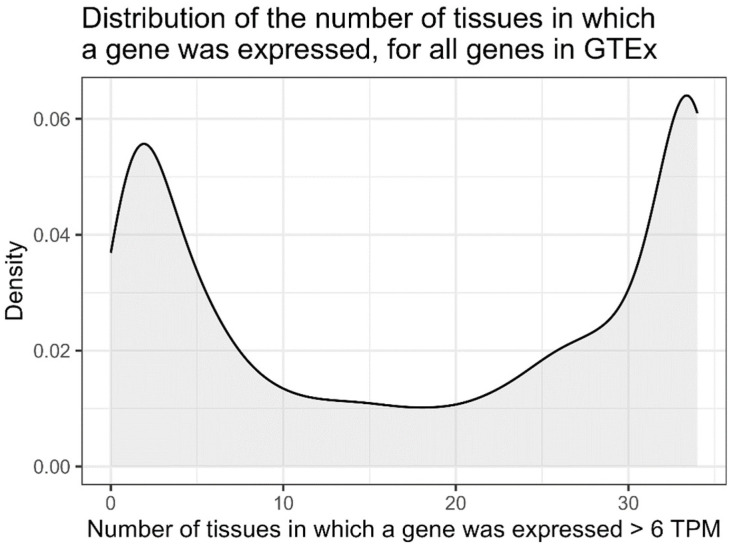
Bimodal distribution of the number of tissues in which a gene could be detected at TPM of at least 6 (summary data for all the genes).

The number of GTEx tissues with at least 100 samples was 34 and the number of genes with TPM expression of at least 6 was, on average, 9888 per tissue. The number of samples per tissue ranged from 103 to 728 (Supplementary Table S1). The distribution of the mean absolute value of the correlation coefficient (calculated for each gene in each tissue) in all tissues is shown in Supplementary Table S2 and illustrated with examples in Figure 2. The distribution of the number of tissues in which a given gene was expressed was bimodal, with a majority of genes expressed at sufficient levels in only few tissues (1–5 tissues) or in almost all analysed tissues (Figure 3).

### 3.2. GTEx Correlations in Tissues—Genes That Were Present in More than n Tissues

Among least correlated genes in GTEx, across tissues, were *RPL13P12* (Ribosomal Protein L13 Pseudogene 12), *NPIPA5* (Nuclear Pore Complex Interacting Protein Family Member A5), *RPSAP58* (Ribosomal Protein SA Pseudogene 58), *RN7SL2* (RNA Component of Signal Recognition Particle 7SL2), and *HBB* (Haemoglobin Subunit Beta). A list of the top 10 most INDIE genes present (TPM ≥ 6) in at least half of the analysed tissues can be found in Table 1.

**Figure 4:**
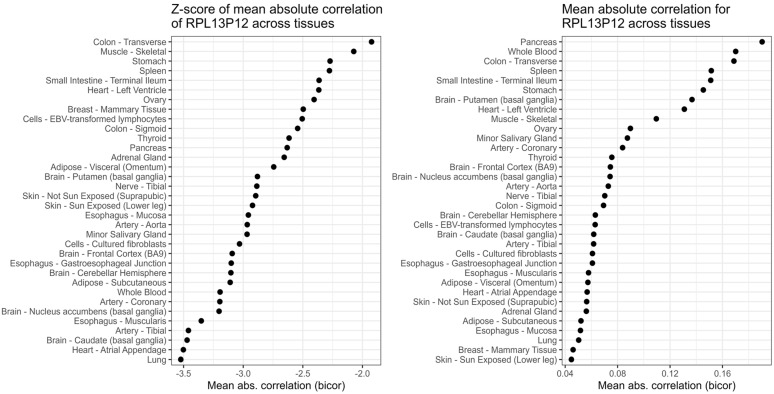
Mean absolute correlations of *RPL13P12* with all other genes across GTEx tissues represented as Z-scores (**left**) and correlation coefficients (**right**) show that the expression of *RPL13P12* was highly independent of most other genes.

The most INDIE gene as reflected by the highest percentage of tissues, where r met the definition, was *RPL13P12*. We present mean absolute correlations and their Z-scores across all tissues in Figure 4. Both measurements demonstrate that the expression of *RPL13P12* was independent from other genes in all of the analysed tissues.

### 3.3. Partial Replication of INDIE Genes in the Whole Blood Across Four External Datasets

Results from GTEx were independently validated in the whole blood and the mucosa of the terminal ileum. For validation in the blood, four datasets were used (E-MTAB-11349, E-MTAB-13397, E-MTAB-12993, E-MTAB-10022). For validation in the terminal ileum, the CEDAR and RISK cohorts were used (E-MTAB-6667, GSE57594).

From the IBD-Character dataset [8], whole blood samples from 590 patients were investigated, including 289 women and 301 men. The study included 156 patients with Crohn’s disease (CD) and 167 with ulcerative colitis (UC), and 267 formed a control group of both healthy and symptomatic individuals without IBD. Mean age among the patients was 34 ± 14 years (median, 31; 1st–3rd quartiles, 23–42 years). For this dataset we set the threshold of median expression at 6 or more, with 8830 genes fulfilling this criterion. Results from whole blood in GTEx were replicated with a within-dataset Z-score ≤ −1.645 for the following genes: *C1QA*, *C1QB*, *ABCB6*, *HIST1H2AD*, *HIST1H2BD*, *TMEM176B*, *TMEM176A*, *SESN3*, *RNASE3*, *OLFM4, RNASE3, CEBPE*, *RETN*, and *PI3*. Several other genes were also quite close to being validated, the most interesting among them being *ZBTB16* (Z-score −1.45; the interest is related to replication in the ileum).

From the migraine dataset by Kumar et al. (E-MTAB-13397), blood samples from 104 patients, including 66 women and 38 men, were analysed. Sixty of them were healthy controls and forty-four had interictal migraine. The mean age was 27 ± 4 years (median, 26; 1st–3rd quartiles, 24–28 years). A total of 8963 genes had a median expression of at least 6. In this dataset, 29 INDIE genes from GTEx whole blood were further validated (Z-score ≤ −1.645), such as *TMEM176B*, *HIST1H3H*, *TMEM176A*, *DEFA3*, *PRDX6*, *ITGA2B*, and *PI3*. Five genes were replicated both in the migraine dataset and in IBD-Character: *TMEM176A*, *TMEM176B*, *PI3*, *HIST1H2BD*, and *HIST1H2AD*.

From the dataset by Rosenheim et al. [13] (E-MTAB-12993), blood samples from 470 participants were obtained, including 129 women and 341 men. Among them, 210 were healthy, and 260 had ongoing SARS-CoV-2 infection. The mean age of the patients was 22 ± 3 years, (median, 22; 1st–3rd quartiles, 19–24 years). There were 12,193 genes with expression equal to or above 6. Four results from GTEx whole blood were replicated: *TMEM176B*, *HIST1H2AD*, *GPX1*, and *SLC2A1*.

In the dataset by Gupta et al. [12] (E-MTAB-10022), 344 patients were included: 246 women and 98 men. Of those, 116 were healthy individuals forming a control group, and 228 were infected with SARS-CoV-2. The population in this study was ethnically diverse: there were 228 patients of Caucasian descent, 70 of Asian descent, and 32 of African descent. The average age was 38 ± 12 years (median, 36 years; 1st–3rd quartiles, 27–47). We identified 9341 genes with median expression equal to or greater than 6 (66% of INDIE genes from GTEx were expressed at sufficient level). In this dataset, seven INDIE genes were validated: *HIST1H2AD*, *TMEM176A*, *TMEM176B*, *GPX1*, *PI3*, *C1QA*, and *TCN1*. All the results from validation are presented in Supplementary Table S3. In Table 2 we present six least correlated genes in GTEx whole blood that were validated in at least two of the external datasets.

### 3.4. Partial Replication of INDIE Genes in the Terminal Ileum in Two External Datasets

In the analysed subset of the CEDAR dataset [14] (E-MTAB-6667), 195 mucosal biopsies of terminal ileum were taken from 182 patients (13 patients were sampled twice): 99 women and 83 men, all of whom were healthy controls. Thirty-three participants had history of smoking. The average age was 53 ± 13.6 years (median, 54 years; 1st–3rd quartiles, 45–63). There were 9834 genes with median intensity-derived expression (log2-transformed offset intensity) equal to or above 6. Five genes were validated despite the dataset utilising microarray technology for expression profiling, and despite analysing mucosal biopsies (CEDAR) instead of full-thickness biopsies (GTEx): *IL1RL1*, *ZBTB16*, *ACAT2*, *PDE4DIP*, *ERLIN2*.

In the dataset by Haberman et al. [15] (GEO GSE57945), terminal ileum mucosa was biopsied in 322 paediatric patients with IBD: 135 female and 187 male. The diagnosis was Crohn’s disease in 218 children and ulcerative colitis in 62. Forty-two participants were found not to have IBD. The average age was 13 ± 3 years (median 12, 1st–3rd quartiles, 10–15). Out of 39,376 genes profiled, 9880 had a median expression level equal to or higher than 6. In the RISK cohort dataset generated by Haberman et al., the results were replicated for 59 genes, despite using mucosal biopsies (Haberman et al.) instead of full-thickness biopsies (GTEx). The top replicated genes included *RPS4Y1*, *RN7SL2*, *PITX1*, *TPSD1*, *TPSAB1*, and *HBB*.

Despite the fact that full-thickness specimens were included in GTEx, we attempted validation of results for the ileum in mucosal biopsies from CEDAR (microarray) and Haberman et al. (RNA sequencing). In the terminal ileum, only the *ACAT2* gene was validated in both datasets simultaneously, but this could be explained by the differences in methodologies utilised in both datasets. For instance, expression of *IL1RL1* and *ZBTB16* was insufficient in the RISK cohort in Haberman et al. However, a number of other genes were replicated in Haberman et al., which differed in transcriptome assessment technology and patient population, with inflammation, which may promote a greater level of correlation between genes involved in mucosal immunity, leaving some other genes with lower Z-scores. Among the validated genes, there were *HBB* and *RN7SL2*, which are among the top 10 cross-tissue INDIE genes from GTEx. *HBB* was not replicated in CEDAR, but it was not far from meeting the threshold (Z-score −1.44), and its mean absolute correlation was even lower for CEDAR (r = 0.07) than for Haberman et al. (r = 0.12) or GTEx (r = 0.18). *RN7SL2*’s expression in was not high enough in the CEDAR dataset to be included in the validation. Results from attempted validation of GTEx INDIE genes in CEDAR and RISK can be found in Supplementary Table S4. It should be underscored that the number of datasets used for validation in the terminal ileum was two.

### 3.5. Other Remarks

For additional analyses that required a longer list of genes, we selected genes validated in blood and ileum datasets, as well as genes from GTEx that met the following criteria: they were expressed at more than 6 TPM in more than six tissues and had correlation coefficient r below 0.2. Expression in more than six tissues was required as there were six tissue types belonging to the nervous system alone. The obtained list of 122 genes was analysed using NDEx Integrated Quarry [18,19,20,21,22], and 10 of the genes were found to be connected with the molecular network responsible for the induction of the angiogenesis.

The extended list of 122 genes was also intersected with the list of housekeeping genes curated by Hsiao et al. [23], and it showed no enrichment for housekeeping genes among INDIE genes. Moreover, some well-known housekeeping genes (*ACTB*, *B2M*, *HPRT*) showed a relatively high mean bicor r > 0.5 in GTEx whole blood. *PRDX6*, however, was an INDIE gene additionally characterised by low variance in the whole blood in GTEx. Moreover, nuclear-encoded mitochondrial genes were broadly expressed but not INDIE.

## 4. Discussion

We investigated GTEx, a landmark transcriptomic dataset, to highlight genes which are characterised by independent expression patterns across tissues. We showed that despite a relatively high level of expression, many genes demonstrated no or little correlation with other genes expressed in a given tissue and that this could generalise to multiple tissues for a single gene. Furthermore, some of the main findings were replicated in external whole blood and ileum gene expression profiling data. Many of the pinpointed genes were specific for cellular types constituting a minority within a given tissue, which may have implications for purity assessment, differential expression analysis, biomarker discovery, and the reliability of deconvolution. Some of the genes with high cellular specificity appear isolated enough in their expression patterns to evoke distinct regulatory mechanisms.

### 4.1. Distributions of Correlations Differ Between Tissues

As shown in Figure 3, the distribution of mean absolute correlations of genes in different tissues from GTEx varied. In most of the tissues, genes below the r < 0.2 correlation cutoff were few. In tissues where this was not the case, the distribution range was narrow. In those tissues, ascertaining which genes were inter-correlated and which were INDIE was more difficult. Quality issues regarding this aspect should be considered, but the samples were filtered for sufficient RNA integrity and expression levels. Nonetheless, the results may not fully generalise to healthy tissues, as in GTEx, the biopsies were taken post-mortem. Many of the tissues available in GTEx cannot be obtained from a healthy volunteer, and the results from tissues that are available have been replicated in datasets generated from live tissues [8,12,13,14,15] and also, in a narrow scope, in this study.

An example of a biological process shaping correlation structures across tissues and populations is provided by mitochondrial genes. Just like the brain may require upregulation of a specific type of fatty acid metabolism compared with other tissues and this may depend on functional context [24], individuals can largely differ in several aspects of mitochondria-related gene expression between themselves (also possibly in relation to activity or environment) [25]. This phenomenon highlights gene expression independence itself as a mechanistic basis for observation of different correlations maps, as opposed to cellular heterogeneity of tissues.

### 4.2. Uncorrelated Genes Within GTEx

Overall, very few genes were systematically INDIE across tissues. Among the genes that met the criteria for analysis, there was only one gene that met our INDIE definition in all 34 tissues. Therefore, we cannot propose any other genes as potentially INDIE regardless of human cell type. This result also suggests that the lack of correlation stems at least partially from tissue specificity. Below we describe a few select genes that were INDIE in the most tissues.

*RPL13P12*, Ribosomal Protein L13 Pseudogene 12, is an INDIE gene in all tissues from the GTEx dataset. As of now, its function and role in human health is mostly unexplored. Nousbeck showed that *RPL13P12* is upregulated in infants (7–12 month old) with Atopic Dermatitis [26]. In a study by Yekula et al., *RPL13P12* was enriched in serum-derived extracellular vesicle RNA from glioblastoma patients responding to dacomitinib [27], partially strengthening the argument that INDIE genes may be more easily included in biomarkers. Furthermore, Yang et al. built a model for predicting response to chemotherapy in metastatic colorectal cancer that uses *RPL13P12* as one of 22 transcripts. However, bringing these scarce pieces of information together into a biologically meaningful framework is not yet possible. *RPL13P12* is a pseudogene of a large ribosomal protein (60S), and as such it could be related to the process of protein synthesis. Yet, as a pseudogene, the original function of the structure of its protein was likely lost, so it is either an evolutional remnant or it might have taken on a regulatory role. The most well-known pseudogene expressed broadly in various tissues is *PTENP1*, which has been shown to help regulate the expression of the original gene, *PTEN*, by intercepting miRNA targeting *PTEN* transcripts. Considering that *RPL13P12* is expressed in many human tissues regardless of other genes, we can speculate that it may also be functionally active. Pseudogenes may fulfil functional roles, but they can also be transcribed simply because of the promoter located in their genomic region [28]. In this case, a gene with expression independent of other genes may serve reference or control roles or be potentially more closely associated with the environment. The expression of *RPL13P12* was not detected in the single-cell GTEx dataset, and the results were not validated in the external datasets within this study, because its expression in the blood and the ileum was not high enough. Searching with BLAST(v 2.17.0) for sequences similar to *RPL13P12* in other species revealed that *RPL13* gene was prone to forming pseudogenes. The expression of *RPL13* (the functional gene) did not show a similar lack of correlation in expression across tissues. This may (but does not necessarily have to) suggest that *RPL12P13* is not tied to regulating *RPL13* expression like *PTENP1* does for *PTPN*. Several enhancer-like signatures, including one placed inside the *RPL13P12* gene, were present, suggesting that proximal and distal enhancers may be at least partially responsible for *RPL13P12*’s broad, cross-tissue expression. Alignment issues could also explain why *RPL13P12* transcripts appeared in every tissue given how many pseudogenes *RPL13* gene has. The *RPL13P12* sequence is nearly identical to *RPL13*, which hints at possible alignment issues and potential biological roles of *RPL13P12* in regulating *RPL13*. Independent of the role of *RPL13P12*, the fact that it systematically did not correlate with other genes, but was broadly expressed, may be worth attention in two aspects. Firstly, its regulation may be of interest, even if it would prove related to cell death. Secondly, its independent expression might be used for technical purposes, depending on external validation.

### 4.3. INDIE Genes Replicated in the Whole Blood

Two of the genes that provide such validation, in the whole blood, are *HIST1H2AD* (Histone Cluster 1 H2A Family Member D) and *TMEM176B* (Transmembrane Protein 176B). *HIST1H2AD* is a canonical histone gene, which is characterised by constant expression. In a study by Sanij et al., *HIST1H2AD* was considered a constitutively expressed gene to analyse the function of Pol I and Pol II in murine fibroblasts [29]. *HIST1H2AD* was overexpressed in MK2 cells undergoing infection with the monkeypox virus [30]. The gene was also implicated in syndromic metopic synostosis [31].

*TMEM176B* is a transmembrane protein, for which most data point towards the immune functions. Single-cell data from the UCSC cell browser indicate that the expression of *TMEM176B* is characteristic for monocytes expressing *CD14* and *CD16* (intermediate monocytes). Vicotria et al. demonstrated that a gene closely related to *TMEM176B*, which was partially validated, *TMEM176A*, is a potential therapeutic target for tumour suppression [32]. It functions as an ion channel for calcium ions but also modulates intracellular signalling by interacting with immune receptors [33]. Both *TMEM176B* and *TMEM176A* were expressed broadly—in 31 out of 34 analysed tissues—but they were INDIE in only few of them, despite successful external replication in the whole blood transcriptomes. Based on this evidence, we suggest that *HIST1H2AD* and *TMEM176B* may be considered INDIE in the whole blood, for new applications that may arise.

Two other genes validated in external datasets are *GPX1* (Glutathione Peroxidase 1) and *PI3* (Peptidase Inhibitor 3). *GPX1* encodes a key gene of oxidative stress management, and it is also a potential drug target in hypertension and metabolic-associated fatty liver disease [34,35]. *PI3* has antibacterial and anti-inflammatory properties, and it has been proposed as a biomarker for gastric cancer chemotherapy prediction [36].

### 4.4. INDIE Genes Replicated in the Ileal Tissues

In the ileal datasets, double external replication was found for only one gene, *ACAT2* (acetyl-CoA Acetyltransferase 2), which encodes a product involved in lipid metabolism. *ACAT2* was shown to both promote and inhibit cancer progression, depending on the condition and specific setting; for example, its upregulation in gastric cancer increases the proliferation rate of the cancerous cells and the likelihood of metastasis [37].

Other genes with the strongest validation in the ileal mucosa (microarray dataset) included *IL1RL1* (Interleukin 1 Receptor-Like 1) and *ZBTB16* (GeneCards Symbol: ZBTB16 (Zinc Finger and BTB Domain Containing 16)). Their co-occurrence in this shortlist is striking, because both are highly implicated in IBD. *IL1RL1* encodes a receptor for interleukin 33, affecting such crucial downstream targets as *IRAK1* and *IRAK4*. *IL1RL1* variants are associated with increased risk of IBD, and one of its variants regulates *sST2* expression induced by corticosteroids in ulcerative colitis [38,39]. However, the gene itself is located in a broader cluster of inflammation-related genes. *ZBTB16* is associated with gut microbial diversity [40]. *ZBTB16* also contains a site differentially methylated in Crohn’s disease [41,42]. This suggests that leukocyte admixtures may influence the ileal transcriptome in ways that are mostly unrelated to mucosal gene expression (which does not exclude epithelial–immune crosstalk).

In the RNA sequencing dataset (Haberman et al.), the most significant validation was found, amongst other genes, for *HBB*. The expression of *HBB* is not limited to the bone marrow; there are data supporting active *HBB* expression in erythrocytes (23). Even if it is relatively low, the large number of erythrocytes may make it noticeable, and *HBB* expression was for many years considered an undesirable artefact in RNA sequencing, leading to the introduction of haemoglobin transcript depletion methods in whole blood samples. Thus, haemoglobin and leukocyte gene expression indicates that cellular admixtures may be present in other tissues in a way that is detectable through transcriptomic analysis. While it may be considered a contamination, for other purposes it could prove useful, e.g., to quantify the level of tissue hyperaemia or the presence of blood vessels, supporting deconvolution. There is a limitation, however, as some organs may unexpectedly express haemoglobin, e.g., the brain [43,44,45].

Another transcript which was validated in ileal mucosa (Haberman et al.) is *RN7SL2*, RNA Component Of Signal Recognition Particle 7SL2. *RN7SL2′s* effect on human health has not been broadly researched, but it is one of the most differentially expressed genes in pancreatic cancer [46]. It has also been shown to increase in paediatric Crohn’s disease regardless of disease behaviour [47].

### 4.5. Main Correlation Coefficients in Transcriptomics

Pearson’s correlation is the most commonly used method of detecting linear relationships between datasets. Because it utilises the means for both datasets, it is best for continuous and normally distributed data, with a mean that represents the centre. This makes Pearson’s correlation coefficient more susceptible to outliers. Spearman’s rank correlation is, as opposed to Pearson’s correlation, non-parametric and thus does not assume normal distribution, relying instead on the fact that data are monotonic, increasing or decreasing consistently. However, Spearman’s correlation may be affected by lower sensitivity. In contrast, bicor retains reasonable sensitivity and still works well even if the data are not distributed normally and also for some non-linear relationships. Bicor also adjusts for the presence of the outliers and handles the data variability well. For this reason it was extensively used in transcriptomics research and also employed in this study.

### 4.6. Limitations

The capacity of correlative analysis to accurately detect real relationships depends on the sample size [48], and at 100 samples the correlation can be as low as 0.2 and still be detected as statistically significant. It is widely accepted that such low correlations are not practically important, with some exceptions under specific conditions, e.g., population-scale epidemiology studies. Our selection of a threshold of r < 0.2 was arbitrary but also relied on expert opinions from the literature. An INDIE gene, as per our definition, may still belong to a small cluster of co-expression. Moreover, while non-linear relationships may be important in the transcriptomic data, they were not considered. Determination of optimal r (or Z-score) cutoff for maximum biological significance would require assessment of a broad range of candidate values through trial and error.

While for GTEx we analysed 34 tissues, we conducted external validation for two, the whole blood and the terminal ileum. This limitation is caused by the paucity of datasets for various tissues, which often cannot be sampled from healthy humans. Validation in more tissues would be beneficial for the analysis, though the results obtained for the validated tissues already provide insight necessary for further research. In future analyses it could prove advantageous to include for validations the datasets from ENCODE or Human Protein Atlas. Similarly, the differences in patterns between transcriptomic and proteomic data may be worth exploring. Furthermore, integrating heterogenous single-cell RNA-seq datasets into the analyses is another good future direction—it would provide greater resolution and allow for estimating the impact of cell-type specific effects. Comparison of gene expression correlation on tissue and cellular levels could potentially provide new insights. The datasets for the terminal ileum, Haberman et al. and CEDAR, differ in methodology; the former was obtained with next-generation sequencing (in children) and the later with microarray. Sequencing and microarray reflect dynamics of RNA abundance differently, which may affect correlation structures (especially linear ones), and they vary in sensitivity to sources of noise and bias. Surprisingly, despite this difference, one gene was validated across both datasets (*ACAT2*). As microarrays hybridise to already-known sequences, their detection range is narrower, and they disallow detection of very high expression levels (oversaturation). The lower sensitivity of microarrays may explain why *RN7SL2* was not detected and validated in the CEDAR dataset. On the other hand, *ZBTB16* and *IL1RL1* did not reach sufficiently high expression levels in the Haberman et al. (RISK) dataset. This is a practical example of how methodology differences could have influenced the results. However, differences in health conditions or even methodology between the datasets increased the specificity of the obtained validation (at the cost of sensitivity). In this context it must be added that the results might have been influenced by the alignment method or RNA preservation artefacts. Analysing genes with TPM ≥ 6 and samples with RIN > 7 was our way to reduce such risks. TPM normalisation was chosen over other data representations because of its improved ability to reflect within-sample gene expression ratios, which are crucial for correlation calculation. Finally, we reiterate that this study analysed bulk tissues, and its conclusions mostly concern work with bulk transcriptomic data.

## 5. Conclusions

A set of genes exhibiting independent expression patterns across various tissues of GTEx was described. Results for each tissue are made available. A considerable share of the findings are readily explained by tissue heterogeneity. However, some results, including those for the monocyte gene *TMEM176B*, may point towards interesting mechanisms of gene expression regulation.

## Figures and Tables

**Table 1:** Top 10 genes with the highest percentage of GTEx tissues yielding INDIE replication (includes only genes with sufficient expression level in at least half of all analysed GTEx tissues).

Gene	Name	% of Validation Across Tissues	*n* of Tissues in Which Gene Was INDIE	*n* of Tissues in Which Gene Had TPM ≥ 6
*RPL13P12*	Ribosomal Protein L13 Pseudogene 12	100%	34	34
*NPIPA5*	Nuclear Pore Complex Interacting Protein Family Member A5	100%	23	23
*AC018738.2*	-	86%	18	21
*RN7SL2*	RNA Component of Signal Recognition Particle 7SL2	85%	29	34
*RPL24P4*	Ribosomal Protien Large 24 Pseudogene 4	84%	16	19
*RPSAP58*	Ribosomal Protein SA Pseudogene 58	82%	27	33
*MTND4P12*	Mitochondrially Encoded NADH:Ubiquinone Oxidoreductase Core Subunit 4 Pseudogene 12	79%	23	29
*ACTA1*	Actin Alpha 1	79%	15	19
*HBB*	Haemoglobin Subunit Beta	78%	25	32
*HBA2*	Haemoglobin Subunit Alpha 2	78%	21	27

INDIE—independent in expression; TPM—transcript per million.

**Table 2:** Least correlated genes in GTEx whole blood which were validated (grey background) in at least two external datasets. *TMEM176B* and *HIST1H2AD* were replicated in each of the four whole blood datasets. Moreover, *HIST1H2BD* was replicated in both datasets in which it was expressed at a sufficient level. *TMEM176A*, *GPX1*, and *PI3* were validated in three out of four datasets, and *C1QA* in two out of three datasets. *CEBPE*, *E2F2*, *RNASE3*, *FTH1P8*, *FTLP3*, and *FAXDC2* were validated in only one dataset, but they were also expressed at a sufficient level in only one dataset.

	*n*	*n*	Z-Score of Mean Absolute Value of r (Bicor) in Datasets			
Gene	datasets	validations	IBD Character	Migraine	COVID-19 d.1	COVID-19 d.2
*HIST1H2AD*—Histone Cluster 1 H2A Family Member D	4	4	−1.90	−2.29	−2.11	−2.67
*TMEM176B*—Transmembrane Protein 176B	4	4	−2.77	−3.60	−1.95	−2.22
*GPX1*—Glutathione Peroxidase 1	4	3	−0.42	−3.02	−2.09	−2.73
*PI3*—Peptidase Inhibitor 3	4	3	−2.53	−2.80	−1.02	−2.70
*TMEM176A*—Transmembrane Protein 176A	3	2		−3.49	−1.44	−2.30
*HIST1H2BD*—Histone Cluster 1 H2B Family Member D	2	2	−1.97	−2.32		

## Data Availability

The source data are available at indicated repositories (Methods Section).

## Supplementary Materials

The following supporting information can be downloaded at https://www.mdpi.com/article/10.3390/genes16091067/s1. Table S1: The number of samples per tissue in GTEx dataset; Table S2: Distribution of mean absolute correlation rates across different tissues from GTEx dataset; Table S3: Validation of uncorrelated genes from GTEx whole blood, in four external datasets; Table S4: Validation of uncorrelated genes from GTEx terminal ileum, in two external datasets.
